# A comparative study of CNN-capsule-net, CNN-transformer encoder, and Traditional machine learning algorithms to classify epileptic seizure

**DOI:** 10.1186/s12911-024-02460-z

**Published:** 2024-03-01

**Authors:** Sergio Alejandro Holguin-Garcia, Ernesto Guevara-Navarro, Alvaro Eduardo Daza-Chica, Maria Alejandra Patiño-Claro, Harold Brayan Arteaga-Arteaga, Gonzalo A. Ruz, Reinel Tabares-Soto, Mario Alejandro Bravo-Ortiz

**Affiliations:** 1 Departamento de Electrónica y Automatización, Universidad Autónoma de Manizales, Manizales, 170001 Caldas Colombia; 2 Departamento de Sistemas e Informática, Universidad de Caldas, Manizales, 170004 Caldas Colombia; 3 Facultad de Ingeniería y Ciencias, Universidad Adolfo Ibáñez, Santiago, 7941169 Chile; 4 Center of Applied Ecology and Sustainability (CAPES), Santiago, 8331150 Chile; 5 Data Observatory Foundation, Santiago, 7510277 Chile; 6 Centro de Bioinformática y Biología Computacional (BIOS), Manizales, 170001 Colombia

**Keywords:** Capsule-Net, Electroencephalograms, Epilepsy, Machine learning, Transformer Encoder

## Abstract

**Introduction:**

Epilepsy is a disease characterized by an excessive discharge in neurons generally provoked without any external stimulus, known as convulsions. About 2 million people are diagnosed each year in the world. This process is carried out by a neurological doctor using an electroencephalogram (EEG), which is lengthy.

**Method:**

To optimize these processes and make them more efficient, we have resorted to innovative artificial intelligence methods essential in classifying EEG signals. For this, comparing traditional models, such as machine learning or deep learning, with cutting-edge models, in this case, using Capsule-Net architectures and Transformer Encoder, has a crucial role in finding the most accurate model and helping the doctor to have a faster diagnosis.

**Result:**

In this paper, a comparison was made between different models for binary and multiclass classification of the epileptic seizure detection database, achieving a binary accuracy of 99.92% with the Capsule-Net model and a multiclass accuracy with the Transformer Encoder model of 87.30%.

**Conclusion:**

Artificial intelligence is essential in diagnosing pathology. The comparison between models is helpful as it helps to discard those that are not efficient. State-of-the-art models overshadow conventional models, but data processing also plays an essential role in evaluating the higher accuracy of the models.

## Introduction

Epilepsy is a neurological disorder characterized by generating discharges in the nervous system without an external stimulus cause which produces convulsions or unusual behavioral moments and sometimes loss of consciousness, affecting people of all ages and geographical locations. It is a common but stigmatized disease, making its diagnosis and treatment challenging, especially in low-resource countries, and increasing mortality rates compared to developed countries [1]. This condition encompasses four main classes: focal, generalized, focal generalized, and unknown. It should be highlighted that recent studies have shown that epilepsy is not just seizures; patients can also experience neuropsychiatric and neurobehavioral symptoms [2]. The symptoms of a seizure can vary widely. Some people with epilepsy only stare briefly during a seizure, while others constantly move their arms or legs [3].

Epilepsy diagnosis and treatment pose unique challenges, especially in low-resource countries where stigma and lack of access to specialized care increase mortality rates. The interpretation of the electroencephalogram (EEG), crucial in diagnosis, is an intensive and variable process dependent on the specialist’s experience [4]. In this context, artificial intelligence (AI) and deep learning are promising solutions, particularly methods based on convolutional neural networks (CNN) that promise to analyze EEG data with greater precision and efficiency [5]. Despite advances in AI for the diagnosis of epilepsy, there is a significant gap in comparing different deep learning architectures with traditional machine learning techniques, which is crucial for identifying the most effective models. This study aims to fill this gap by comparing CNN-based methods and traditional machine learning techniques, seeking to improve the accuracy and efficiency of epilepsy diagnosis. The findings of this research could transform the diagnosis of epilepsy, offering faster and more precise methods and reducing the economic and social burden of this condition, especially in regions with limited access to neurology specialists.

In machine learning mechanisms, hyperparameters are adjusted [6]. A pipeline mechanism is used to modify these hyperparameters. It aims to chain together different steps in an organized manner to extract features and make adjustments to a model. Following this, a grid search is employed. This technique explores the best values and evaluates the model’s performance for each combination of values [7].

In recent years, new deep-learning mechanisms have improved the model’s capacity. Suat Toraman discusses Capsule Neural Networks (Capsule-Net) and their role in enhancing the performance of image prediction models. Additionally, Toraman proposes a Capsule-Net model for predicting epileptic seizures along with 1D convolutional networks [8]. More recently, Shuaicong Hu et al. proposed a hybrid transformer model for classifying epileptic seizures, which primarily consists of four blocks: Rhythm Embedding, Positional Encoding, Self-Attention, and Classifier [9].

Capsule-Net are a new type of machine learning (ML) architecture recently developed to overcome the disadvantages of CNNs. Capsule-Net is resistant to affine rotations and translations, which is useful when dealing with medical image datasets. In addition, Vision Transformer (ViT) based solutions have recently been proposed to solve the long-term dependency on CNNs. Implementation to deep learning models with Capsule-Net and Transformer Encoder offers improvements in performance and computational cost since Capsule-Net requires less training data compared to CNNs and Transformer Encoder models are more robust and have better performance. However, it has yet to be explored in the medical data field [10].

Yi Wei et al. [11] propose a Transformer model and a Capsule-Net to improve performance in emotion recognition, the Transformer model is used to extract information, and the Capsule-Net is used to refine the features, thus avoiding limitations that arise from CNNs, achieving excellent performance in this field of study. The combination of these models served as a motivation for our research.

Considering that the purpose of this paper is to compare models for the classification of electroencephalograms, the main contributions are:

- The Capsule-Net model is modified and applied to signals or flat data, releasing the code to be replicated in other problems.
- State-of-the-art architectures were combined for the creation of new optimized models to achieve the best possible classification in addition to this, it is compared, and a verdict is given as to which of the models is the most efficient for the database used; however, like the modified Capsule-Net model, the repository is published for experimentation on other types of pathologies or with different databases.
- The optimization of the models improves code compilation times, helping to reduce computational costs and giving way to the ease of doing multiple experiments.

This paper is structured into key sections: “Introduction”, which sets the stage by providing a comprehensive overview of the addressed ideas; “Related work”, exploring works related to the current study; “Materials and methods”, offering insights into the methodologies employed; “Results”, presenting the outcomes of the study; “Discussion”, analyzing and interpreting the results; and “Conclusion”, summarizing the key findings and implications. Each section contributes to a comprehensive understanding of the research endeavor.

## Related work

Epilepsy is a chronic brain disease that affects people of all ages. It is estimated that around 50 million people suffer from this disease, making it one of the most common neurological diseases. The World Health Organization estimates that 70% of people with epilepsy can live seizure-free if properly diagnosed and treated, so in recent years, new research has emerged to identify epilepsy using deep learning such as the case study conducted by Gaowei Xu et al. [12]. Gaowei Xu et al. implemented a one-dimensional convolutional neural network model of short-term memory (1D-CNN-LSTM) to analyze epileptic seizures through EEG signals. They initially preprocessed and normalized the data, and then they created the CNN to extract the features from the data that pass to LSTM (Long Short-Term Memory) layers to extract the temporal features to finally introduce these outputs into fully connected layers, thus achieving an accuracy of 99.39% in binary detection and 82% in multiclass detection, demonstrating the potential of deep learning models for epilepsy detection.

On the other hand, Mengnan Ma et al. [13] used a combination of recurrent neural network (indRNN) and 1D CNN to detect periods of interictal, preictal, and ictal epilepsy; thus, the 1D-CNN was used to extract the features of the EEG signal while the indRNN was used to distinguish the categories based on the extraction of features so with this combination a deep learning model was created for the spatiotemporal detection of the disease. In this model, in small sample datasets from the University of Bonn, the proposed method achieved 100% classification accuracy and specificity in detecting all three classes.

Another research carried out by Rubén San-Segundo et al. [14] used an EEG database from Bern-Barcelona [15] and the epileptic seizure recognition database [16]; However, the first contains data from two categories unlike the second, thus dividing into three classifications: healthy (Z), interictal (F) and ictal (S), several transformations of the EEG signal with Fourier, Wavelet and decomposition were evaluated empirically, obtaining various scenarios for the detection of seizures, which generated the best results when using the Fourier transform. Accuracy increased from 99.0% to 99.5% for classifying non-seizure vs. seizure records, from 91.7% to 96.5% when differentiating between healthy, nonfocal, and seizure records, and from 89.0% to 95.7% when considering adjustment, focal and seizure records.

Amirmasoud Ahmadi et al. [17] presented a new algorithm for seizure classification using the wavelet packet transform (WPT) to identify the essential characteristics of the signal better. They used a public database from the Epilepsy Centre at Bonn University, which contained EEG signals from five healthy and five patients with epilepsy. This dataset was divided into 17 subsegments that, in turn, were organized into WP trees. From these coefficients, they used statistical characteristics such as standard deviation (STD) and root mean square (RMS). Then, they used the Support vector machine (SVM) classifier for binary classification in seven cases. The best result was obtained by classifying class A (healthy person with open eyes) versus class E (epileptic seizure) with an accuracy of 99.64%, while for the binary classification of class E versus the remaining four classes, an accuracy of 97.85% was obtained.

Lina Wang et al. [18] initially performed a database treatment at the University Hospital Bonn, Germany, that contained data from 5 healthy patients and five patients with epilepsy, thus filtering the EEG signal with a method that eliminates noise using the wavelet threshold. They analyzed the signals in the time, frequency, and time-frequency domains and performed a nonlinear analysis using empirical modal decomposition (EMD). They implemented five algorithms, including K-nearest neighbors (kNN) and SVM, the latter being the classifier with the highest accuracy since it obtained a value of 99.25% with the nonlinear multi-domain analysis with 10-fold cross-validation and a standard deviation of 0.28.

Shen et al. [19] propose a real-time approach to detecting epileptic seizures using EEG. This approach combines a tunable Q wavelet transform and a CNN. The authors extract spectral and time-domain features from the EEG, such as statistical moments and spectral power, and convert them into image-like data to feed the CNN. The proposed method was evaluated using the CHB-MIT database, and promising results were obtained. The accuracy was 97.57%, with a sensitivity of 98.90% and a false positive rate of 2.13%. In addition, the feasibility of implementing this approach in real-time is highlighted, making it suitable for application in clinical settings for seizure detection.

Finally, a recent study by Chen et al. [20] proposes an automated method for detecting epileptic seizures in EEG signals using a CNN-based classifier and feature fusion and selection. The authors extract mixed features from EEG signals using discrete wavelet decomposition (DWT), including approximate entropy (ApEn), diffuse entropy (FuzzyEn), sample entropy (SampEn), and STD by using a random forest algorithm to select relevant features and applying CNNs to classify epileptic EEG signals. Experimental results from reference datasets, such as Bonn EEG and New Delhi, demonstrate the efficacy of the proposed method. For the Bonn dataset’s interictal and ictal classification tasks, the model achieves an accuracy of 99.9%, a sensitivity of 100%, an accuracy of 99.81%, and a specificity of 99.8%. For the interictal-ictal case of the New Delhi dataset, the model achieves 100% classification accuracy, 100% sensitivity, 100% specificity, and 100% accuracy.

**Table 1:** Comprehensive Overview of Studies in Epilepsy Seizure Recognition

Authors	Method	Subjects	Modalities	Performance	Limitation
Gaowei Xu et al. [12]	CNN-LSTM 1D	Epileptic Seizure Recognition dataset: 500 patients with 5 health conditions.	EEG	ACC on binary: 99.39% ACC on the five class: 82%	The article does not provide interpretability of the model used.
Mengnan Ma et al. [13]	RCNN	Epileptic Seizure Recognition dataset: 500 patients with 5 health conditions. Privated Dataset: 15 patients with 3 health conditions.	EEG	ACC on the three class: 100%	The article uses a database with few patients.
Rubén San-Segundo et al. [14]	CNN-1D	Bern-Barcelona EEG dataset: 5 patients with 2 states. Epileptic Seizure Recognition dataset: 500 patients with 5 health conditions.	EEG	ACC: 98.9% between focal and non-focal signals; 99.5% for classifying non-seizure vs. seizure; 96.5% between healthy, non-focal and seizure; 95.7% when considering healthy, focal and seizure.	The article uses a database with few patients.
Amirmasoud Ahmadi et al. [17]	SVM	Epilepsy Centre at the Bonn University: Five health patients and Five epilepsy patients.	EEG	Performance varies between 94.38% to 99.64% each corresponds to the cases A versus E and D versus E respectively.	The article uses a database with few patients.
Lina Wang et al. [18]	DWT, multi-domain feature extraction and nonlinear analysis	Epilepsy Centre at the Bonn University: Five health patients and Five epilepsy patients.	EEG	ACC: 99.25%	The article uses a database with few patients.
Shen et al. [19]	TQWT and CNN	Database CHB-MIT: 5 males and 17 females	EEG	ACC: 97.57%	The article uses a database with few patients.
Chen et al. [20]	RF + CNN	Epilepsy Centre at the Bonn University: Five health patients and Five epilepsy patients New Delhi EEG dataset: Ten epilepsy patients	EEG	ACC: 99,9%	The study does not mention any limitations related to the generalizability of the proposed model to different patient populations or EEG recording conditions.

This research demonstrates the ability of the proposed approach to detect and classify EEG signals associated with epileptic seizures with high accuracy, which is of great relevance in the clinical detection of epilepsy. These studies are detailed in Table 1.

## Materials and methods

### Database

The epilepsy seizure recognition database [16] consists of 5 individuals and 4097 data points of 23.5 seconds each. This database mixes each data point into 23 fragments with 178 data points per second. It is divided into five classes (a, b, c, d, e):

- Class (a) represents the recording of epileptic activity.
- Class (b) represents the EEG recording from the area where a tumor was present.
- Class (c) represents the healthy part of the brain after tumor localization.
- Class (d) represents the recording of the patient with closed eyes.
- Class (e) represents the EEG recording of the individual with eyes open.

It was decided to divide the database into two ways to compare the results obtained from the study and to have clear and precise information on how to classify the different brain activities corresponding to epilepsy disease. The original dataset has five folders with 100 records each from another patient, totaling 5 individuals/persons. Each file is a recording of brain activity for 23.6 seconds. The corresponding time series is sampled into 4097 data points, but the dataset used was modified, dividing and mixing each data point 4097 into 23 chunks, each containing 178 data points per 1 second. We are leaving; as a result, 11500 pieces of information.

With this in mind, it was decided to split the dataset into two classifications, a binomial and a multinomial classification, looking for the best performance of the model when classifying the different signals.

#### Binary partition

**Fig. 1:**
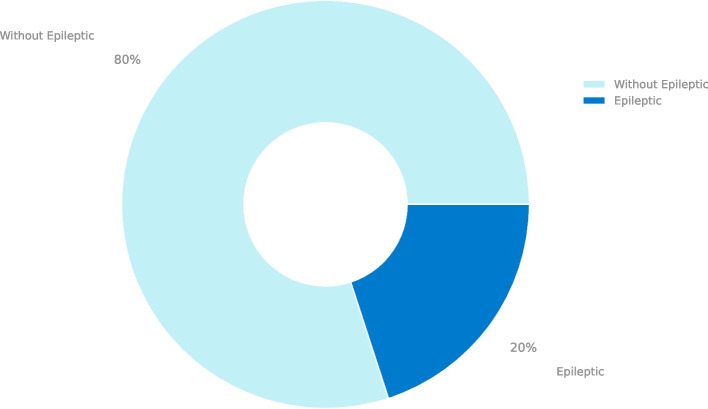
Data ditribution of the binary partition of the database of patients with and without pathology

With this dataset division, the work was divided into two phases: The first phase worked with only two classes in search of the best binomial classification. How the classes were divided in this phase was the following: The first class represented the brain activity when the epileptic seizure occurred (Class 1) with a total of 2300 samples, and the second one represents no epileptic activity (Class 0) with a total of 9200 pieces, in this class are grouped the other four classes of the original dataset, being these four different classes where no epileptic seizure occurred. As shown in the Fig. 1.

#### Multiclass partition

**Fig. 2:**
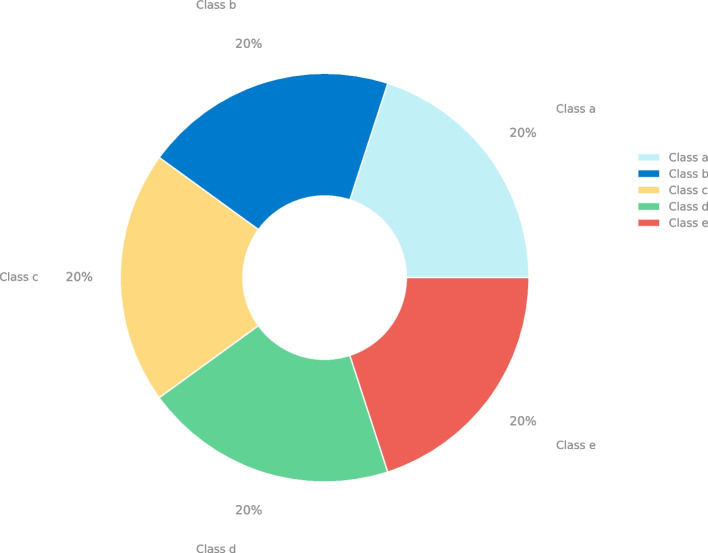
Data distribution of the partition in 5 database classes, alphabetically distributed

The second phase was carried out in search of the best multinomial classification with the five original classes of the dataset. Each Class represents a different moment when the brain activity was recorded, Class (a) represents the recording of epileptic activity, Class (b) represents the EEG recording from the area where a tumor was present, Class (c) represents the healthy part of the brain after tumor localization, Class (d) represents the recording of the patient with closed eyes, Class (e) represents the EEG recording of the individual with eyes open. Each Class has a complete recording of 2300 brain activity samples from the five folders, each with 100 patients.As shown in the Fig. 2.

### Database preparation

As mentioned earlier, the database was divided into two and five classes. For two classes, standard normalization was performed. For five classes, the following preprocessing steps were carried out:

- *Any*:The database is without additional preprocessing.
- *Scaling*: Database with standard normalization.
- *PCA*: Principal Component Analysis (PCA) was performed on the database. It is a technique used to extract the most relevant features by finding the direction of the highest variability of the data, representing the data in a smaller dimension without losing too much information [21]. For this, a standardization process is carried out, followed by a covariance matrix calculation, calculation and selection of vector components, and finally, a data projection. For this database, PCA was performed, which reduced the channels from 178 to 40 in the multiclass models, so the models with and without PCA were compared with the standard scaler.
- *Scaling + PCA*:The database underwent normalization followed by PCA, just as the features were reduced from 178 to 40 when PCA alone was performed.

### Hyperparameters

#### Grid search

When we talk about Grid Search, we are talking about a very common or traditional method for hyperparameter optimization, where a complete search is performed on the subset of data of the space bounded by the same hyperparameter of the model. This is because the parameter used for the model can sometimes include areas with fundamental or unbounded values. One of the big problems with the grid search is the need to apply a specific limit since it suffers in huge dimensional spaces. Still, its great advantage is the ease with which the process can be stopped since the values of the hyperparameters used by the model are independent of each other [22].

#### Pipeline

The term pipeline is used for objects capable of combining estimators and various transformers to create a combined estimator [23]. It is also used to help optimize the data flow to the desired model, including several essential parameters for the proper functioning of the model, such as features, results, predictions, and raw data. The importance would be substantially improved performance and effectiveness, which is fundamental in developing many machine learning models.

This paper used a grid search and pipeline model to find the hyperparameters best fitting the machine learning models. These can be seen in the Table 2.

**Table 2:** Hyperparameters achieved for the machine learning models after grid search and pipelines. We can see the models, the evaluated parameters chosen for both two classes and multiclass, and their descriptions

Algorithms	Hyperparameter	Two-class	Multi-class	Description
DTC	criterion	entropy	gini	Evaluates the quality of a division
	max_depth	None	N/A	Maximum depth of the tree
	min_samples_leaf	4	2	Minimum number of samples required per leaf node
	min_samples_split	10	10	Samples needed to split an internal node
MLP	activation	logistic	relu	Function that activates the hidden layer
	hidden_layer_sizes	(100,)	(100,50)	Number of neurons of the i-th hidden layer
	learning_rate	constant	constant	Learning rate programming for weight updates
	solver	Adam	Adam	Solver for weight optimization
KNN	algorithm	auto	auto	Calculate nearest neighbors
	leaf_size	1	1	Leaf size passed to BallTree or KDTree
	n_neighbors	1	1	Number of neighbors
	p	2	1	Indicates the power for the Minkowski metric
	weights	‘uniform’	‘uniform’	Used in prediction
SGDC	alpha	0.001	0.01	Constant that multiplies the regularization term
	loss	‘hinge’	‘hinge’	
	max_iter	2000	1000	Number of epochs performed on training data
	penalty	‘l2’	‘l1’	Regularization term
	weights	‘uniform’	‘uniform’	Used in prediction
ETC	min_samples_split	4	N/A	Samples needed to split an internal node
	n_estimators	150	300	Number of trees in the forest
	random_state	20	20	Controls the bootstrapping of the samples
	weights	‘uniform’	‘uniform’	Used in prediction
SVM	C	10	10	Regularization parameter
	gamma	‘scale’	‘auto’	Is a coefficient
	kernel	‘rbf’	‘rbf’	Is the type of kernel in use
RFC	max_depth	None	None	Maximum depth of the tree
	min_samples_split	2	2	Samples needed to split an internal node
	n_estimators	500	500	Number of trees in the forest
	random_state	40	40	Controls the bootstrapping of the samples
GB	learning_rate	0.1	0.1	Is a compensation between n_estimators and learning_rate
	max_depth	5	7	Maximum depth of the regression estimators
	n_estimators	200	200	The number of stages to be performed
	random_state	40	10	At each iteration of reinforcement controls the seed

#### Batch normalization (BN)

For data normalization, the BN is used to normalize the features in each data map to have a mean of 0 and a variance of 1, allowing rescaling and retranslating of the distribution. This process in training allows for a higher learning speed [24] (see Eq. 1).

1 $$\begin{aligned} BN(x,\gamma ,\beta ) = \beta + \gamma \frac{{x - E[X]}}{{\sqrt{Var[X] + \varepsilon } }} \end{aligned}$$

Where:

$\gamma$ = The re-scaling scalar.

$\beta$ = re-translation scalar.

*E*[*X*] = expectation.

*Var*[*X*] = variance.

#### Scaled exponential linear unit (SELU)

It was proposed by Klambauer et al. in 2017 [25]; this is a nonlinear function that works linearly as long as the values are positive, but if otherwise, they are negative, it will behave exponentially. This allows the values to scale and propagate around the multiple layers of the neural network using its two constants $\lambda$, which is a value around 1.0507, and $\alpha$, which is the negative slope with an approximate value of 1.67326. Furthermore, this is considered a self-regulating function since, as the information flows through the network, the mean and variance remain stable, helping to improve the stability of the model [26] (see Eq. 2).

2 $$\begin{aligned} \text {{SELU}}(x) = \lambda \left\{ \begin{array}{ll} x &{} \text {{if }} x > 0 \\ \alpha \cdot (\exp (x) - 1) &{} \text {{if }} x \le 0 \end{array}\right. \end{aligned}$$

### Dropout

**Fig. 3:**
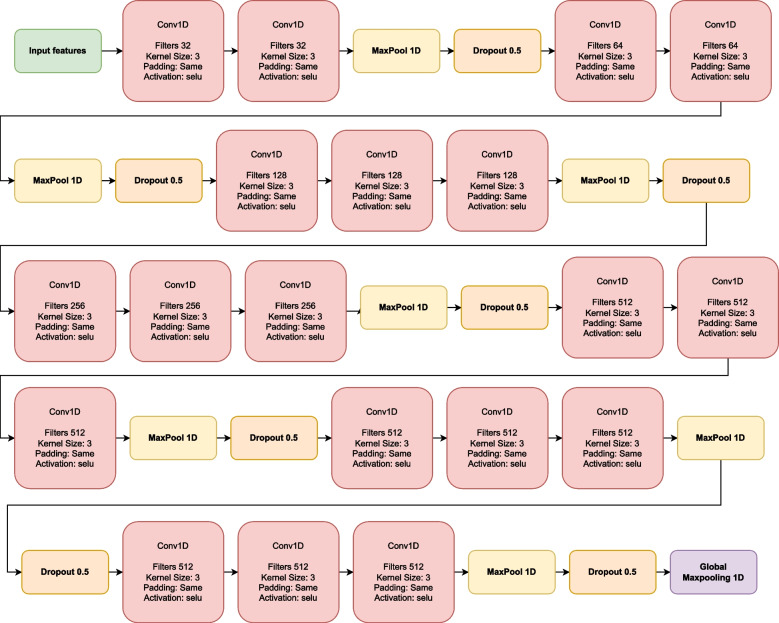
Feature extraction model composed of convolutional layers followed by max pool and dropout. The convolutional layers are distributed: 2 of 32, 2 of 64, 3 of 128, 3 of 256, and finally, 9 of 512 divided into blocks of 3

The regularization method counteracts overfitting by temporarily deactivating randomly selected nodes and their connections. This prevents the neural network from excessively co-adapting and relying too heavily on specific features, limiting its ability to recognize only the training data. Dropout not only addresses overfitting but also contributes to developing more resilient networks. By forcing the network to operate with various samples, Dropout promotes robustness. This approach facilitates the averaging of predictions and reduces test time [27], as highlighted in Fig. 3, to mitigate overfitting in the network.

### Data balanced

#### Synthetic minority over-sampling technique (SMOTE)

We have a very unbalanced data set in the binary classification, so data balancing is performed with SMOTE. This works in such a way that synthesized data can be generated using similar neighboring samples and linear combinations between them; this helps to increase the data of the minority class, allowing the model to learn the patterns of the unbalanced course better [28].

#### Adaptive synthetic sampling (ADASYN)

It is used to create synthesized data from the minority class to balance the data. It focuses on generating synthesized data in feature regions where the minority class examples are few, helping the model better capture the class data with fewer data, and avoiding overgeneralization of the model [29]. Synthetic samples are created by selecting a minority example and randomly choosing some of its neighbors, for which their density is calculated. This is based on interpolation and employs straight-line or k-means techniques. Thanks to the relative density, more examples of the minority class are generated [29].

Table 3 compiles the best hyperparameters of the machine learning models obtained in the GridSearch and Pipeline process for data balancing with SMOTE and ADASYN.

**Table 3:** The best hyperparameters of the machine learning models were obtained for data balancing with SMOTE and ADASYN

Algorithms	Hyperparameter	SMOTE	ADASYN
DTC	criterion	Entropy	Entropy
	max_depth	None	None
	min_samples_leaf	1	1
	min_samples_split	2	2
MLP	activation	relu	relu
	hidden_layer_sizes	(100, 50)	(100, 50)
	learning_rate	constant	adaptive
	solver	adam	adam
KNN	algorithm	auto	auto
	leaf_size	1	1
	n_neighbors	1	1
	p	2	2
	weights	uniform	uniform
SGDC	alpha	0.001	0.001
	loss	hinge	hinge
	max_iter	3000	3000
	penalty	elasticnet	l2
ETC	min_samples_split	2	2
	n_estimators	150	300
	random_state	40	50
SVM	C	10	10
	gamma	scale	auto
	kernel	rbf	rbf
RFC	max_depth	None	None
	min_samples_split	4	4
	n_estimators	150	150
	random_state	30	
GB	learning_rate	0.1	0.1
	max_depth	7	7
	n_estimators	200	200
	random_state	40	40

### Models

#### Machine learning models:

 

- *The Extra Trees Classifier and the Random Forest Classifier (ETC and RFC)*: Are machine learning models that refer to decision trees and are used for classification and linear regression. However, they tend to overfit, which causes problems with new data [30]. The RFC randomly trains multiple decision trees using training data subsets to address this. Finally, a voting algorithm is applied to obtain the best results [31, 32]. The ETC adds randomness to the training process to increase diversity among the trees and improve the model’s performance [33].
- *The Support vector machine (SVM)*: This model can solve linear and nonlinear classification and regression problems. It is particularly well-suited for small and moderately complex data sets. The fundamental concept behind SVM (Support Vector Machine) classification is to separate classes by maximizing the decision boundaries concerning the closest training patterns. Furthermore, it aims to maximize the distance from the nearest training pattern while introducing nonlinearity. SVMs [30] achieve linearly separated classes by utilizing kernel functions that modify or add features based on the training set.
- *Gradient Boosting*: Combines multiple weak learning models into a single robust model [34]. The general idea is that the Gradient Boosting (GB) training process starts with a simple base model and fits it to the training data. Then, the residuals of this first base model are calculated. A new weak model is trained using the residuals as the target in each subsequent iteration. This new model is added to the existing ensemble of models and fitted to the updated residuals [30].
- *The Decision Tree classifier (DTC)*: Is based on a decision tree, which selects the most relevant features or attributes from the training set. In addition to this, additional criteria such as node stopping or pruning can be added to the decision tree [35].
- *KNeighbors Classifier (KNN)*: In the case of the K-NN algorithm, the example data is represented in an n-dimensional space, where n is the number of attributes of the data. Each point in this n-dimensional space is labeled with its corresponding class value. The fact is placed in this n-dimensional space to determine the classification of unclassified data, and the class labels of the k nearest k data points are observed. Typically, k is an odd number. The class that appears most frequently among the k nearest data points is taken as the class of the new data point. In other words, the decision is made by voting on the k neighboring points. One of the significant advantages of this generic K-Nearest Neighbor algorithm for classification discovery is that it lends itself to parallel operations [36].
- *Stochastic Gradient Descent(SGD)*: The SGD is a variant of the Gradient Descent algorithm. Still, unlike the latter, it does not use the entire training data set in each iteration but instead uses mini-batches to calculate the gradient and adjust the model parameters. This decreases the computational burden. In addition to estimating its loss function, hyperparameters such as the learning rate and the number of mini-batches must be adjusted [37].

#### Convolutional neural network

The convolutional neural network is based on the preamble that data have locally important patterns or features that can be extrapolated. There are multiple convolutional neural networks; however, they mostly all follow the same structure. These consist of 3 layers: Convolutional, which aims to learn the input feature representation; this is composed of several convolution kernels that map the different features; these are interconnected first to understand the input and then use the activation function. After this, we have the second layer fully connected, i.e., all neurons from the previous layer are directly related to the next one to generate general semantic information. Finally, we have an output layer for classification tasks that commonly has a softmax activation operator and an optimizer [38].

The following Eq. 3 denotes this:

3 $$\begin{aligned} {M^l} = pool\left( f\left( {norm\left( {\sum \limits _{i = 1}^n {\left( {M_i^{l - 1} * K_i^l} \right) + b_{}^l} } \right) } \right) \right) \end{aligned}$$

Where: $M^l$ represents each of the feature maps. ${M_i^{l - 1}}$ is the pre-feuture map layer, ${K_i^l}$ is the kernel, b is the bias, * refers to convolution.

**Fig. 4:**
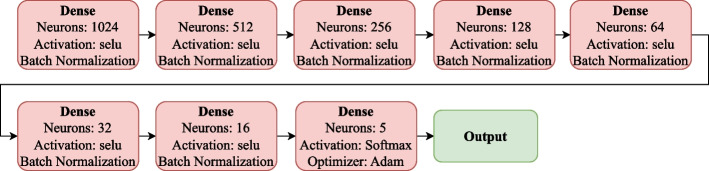
Dense layers are tightly connected with the selu activation method. They are arranged as follows: 1024, 512, 256, 128, 64, 32, 16, 5 of neurons

The neural network we use in this paper is described in Figs. 3 and 4. The 1D convolutional layers (Conv1D) comprise a kernel of size 3, padding same, and a selu activation method. In the output of each set, we have a 1D maxpole and a dropout 0. 5, the sets are ordered in such a way that we have two layers of 32 neurons, 2 layers of 64 neurons, 3 layers of 128 neurons, 3 layers of 256 neurons, and 6 layers of 512 neurons divided into groups of 3. Connected to this last output, we have a Global Max pooling 1D that connects us with our fully connected dense layers. The neural network we use in this paper is described in Figs. 3 and 4. The 1D convolutional layers (Conv1D) are composed of a kernel of size 3, padding SAME, and a selu activation method, and in the output of each set, we have a 1D maxpole and a dropout 0. 5, the sets are ordered in such a way that These have a batch normalization method with a Selu activation method, so we have layers of 1024, 512, 256, 128, 128, 64, 32, and 16 neurons. Finally, we have a classification layer of 5 neurons, one for each class, a softmax activation method, and an Adam learning rate optimizer of 0.001.

#### Capsule-net

**Fig. 5:**
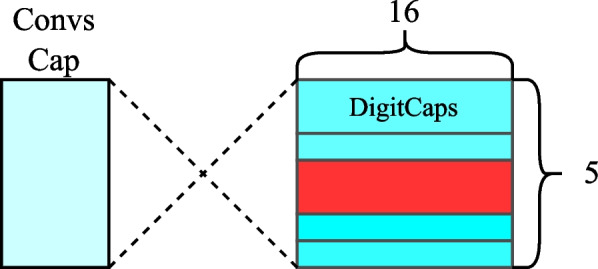
Capsule-Net model. In the input, you have the convolutional layers denoted as conv caps

CNNs have limitations, such as the need for large amounts of training data, the inability to handle ambiguity and changes in object orientation, and the loss of information across layers. To overcome these shortcomings, Geoffrey E. Hinton proposed a new approach known as Capsular Neural Networks (Capsule-Net) described in Fig. 5. Capsule-Net implements groups of neurons called capsules, which encode spatial information and the probability of the existence of an object in an image.

Each capsule represents the instantiation parameters of a specific entity, such as an object or a part of an object. The length of a capsule’s vector indicates the probability that the entity exists, while its orientation represents the instantiation parameters. In Capsule-Net, the model learns to represent an image inversely by examining it and attempting to predict the corresponding instantiation parameters. This is achieved by trying to reproduce the object the model thinks it has detected and comparing it to labeled examples in the training data, thus improving the ability to predict the instantiation parameters.

Active capsules at one level predict the instantiation parameters of higher-level capsules using transformation matrices. When several predictions match, a higher-level capsule is activated. Unlike the max-pooling used in CNNs, Capsule-Net does not lose information about the exact position of the entity within a region, allowing higher-level capsules to cover larger regions of the image [39]. As one moves up the hierarchy, the lower-level capsules encode more basic information, such as simple geometric shapes and their spatial position. In contrast, the more complex capsules represent more structured geometries.

A key feature of Capsule-Net is its ability to handle spatial and hierarchical relationships between entities in an image. Unlike CNNs, where features are combined using convolution and clustering layers, Capsule-Net allows lower-level capsules to interact and predict the properties of higher-level capsules using transformation matrices. This architecture more effectively captures objects’ hierarchical relationships and geometry in an image, resulting in a more robust and complete representation of visual features. It first extracts learned features that are then fed into a fully connected neural network that produces a classification. The network can learn features by chaining convolutional blocks whose layers learn simple features, but as the blocks are usually routed with pooling, they significantly improve the classification by discarding unimportant activations, which makes the classifier robust to small transformations in the input data [40].

The algorithm for the Capsule-Net is the next:

1. procedure ROUTING $$(\hat{U}_{j|i}, r, l)$$
2. for all capsule *i* in layer *l* and capsule *j* in layer $(l+1): b_{ij} \leftarrow 0.$
3. for *r* iterations do:
4. for all capsule *i* in layer $l: C_{i} \leftarrow \texttt {softmax}\left( b_{i} \right)$; where softmax is: $c_{ij} = \frac{\exp (b_{ij})}{\sum _{k}\exp (b_{ik})}$
5. for all capsule *j* in layer $(l+1): s_{j} \leftarrow \sum _{i} c_{ij} \hat{U}_{j|i}$.
6. for all capsule *j* in layer $(l+1): v_{j} \leftarrow \texttt {squash}(s_{j})$; where squash is: $v_{j} = \frac{||s_{j}||^{2}}{1+||s_{j}||^{2}}\frac{s_{j}}{||s_{j}||}$
7. for all capsule *i* in layer 1 and capsule *j* in layer $(l+1): b_{ij} \leftarrow b_{ij} + \hat{U}_{j|i} \cdot v_{j}$ **return** $v_{j}$ where $s_{j} = \sum _{i} c_{ij} \hat{U}_{j|i}$, $\hat{U}_{j|i} = W_{ij} U_{i}$

The vector output $v_{j}$ of capsule *j* represents its resulting output, while $s_{j}$ represents the total input received by that capsule. In layers beyond the initial layer, the total input $s_{j}$ of a capsule is calculated as a weighted sum of the “prediction vectors” $\hat{U}_{j|i}$ from the capsules in the layer below. This is achieved by multiplying the output $u_{i}$ of a capsule in the lower layer by a weight matrix $W_{ij}$. The coupling coefficients $c_{ij}$ play a crucial role in determining the weights and are obtained through an iterative dynamic routing process [39].

The Capsule-Net was incorporated into the model as a subsequent layer to the convolutional and pooling layers, with dimensions adapted to facilitate efficient processing of the capsule vectors. In the first step, an activation function is applied that normalizes and compresses the output values to ensure they are in an appropriate range. This compressed output is passed to the Capsule-Net, where linear transformations generate a tensor in response.

#### Transformer encoder

Transformers consist of an encoder-decoder architecture, where the encoder processes the input sequence and generates a representation, while the decoder generates the output sequence based on that representation. Each encoder and decoder layer of a transformer consists of multiple self-attenuating heads and feed-forward neural networks [41].

The key component of transformers is the attention mechanism, which allows the model to focus on different parts of the input sequence when making predictions. This attention mechanism allows the transformers to capture contextual information from both preceding and following words in a sentence, leading to better understanding and representation of the input data [41].

Adequate transformer performance is due to the use of Attention, which allows the model to focus on the relationship to other words directly related to the text sequence in the input. Transformers are helpful in most NLP tasks, such as linguistic modeling and text classification. There are different structures for different types of problems. The basic coding layer is a standard building block for these architectures, with various specific “heads” to apply depending on the problem being solved.

In the transformer, the Attention module repeats the computation several times in parallel. Each of these is referred to as an attention head. The Attention module splits its N query, key, and value parameters and passes each split independently through a separate header. These similar attention calculations are combined to produce a final attention score. This draws attention from multiple heads and allows the transformer to encode multiple conditions and nuances for each word [42].

**Fig. 6:**
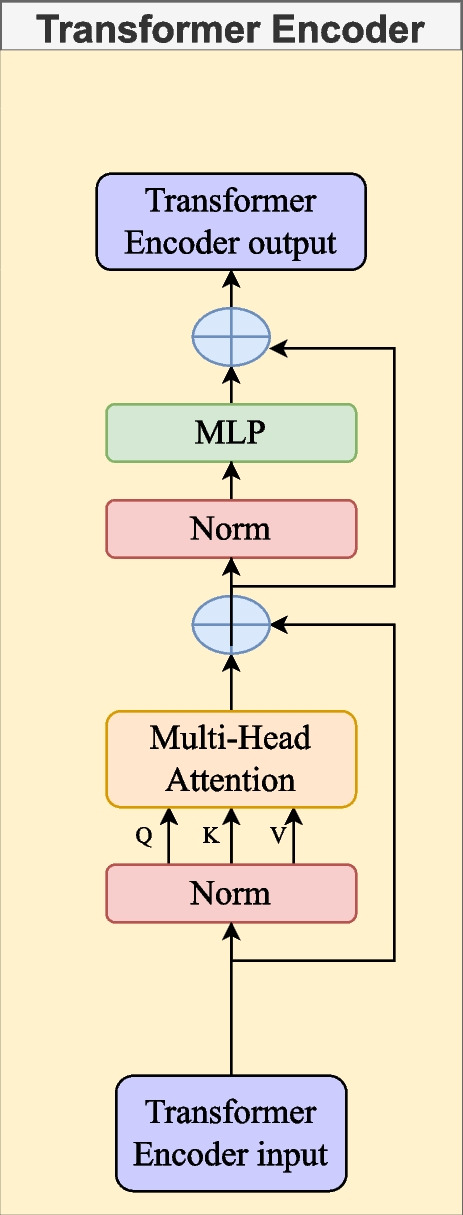
Transformer Encoder. Transformer encoder input is the output of the CNN. The figure contains Multi-Head Attention layers that weigh the relevance of each input vector. The MLP processes these representations of each vector. Residual Connections and Layer Normalization facilitate an efficient and stable flow of information. The output is a deep contextual representation of the input

Given the same set of queries, keys, and values, they were entering the practical application, opting for a model that combines knowledge of different behaviors of the exact attention mechanism to capture dependencies of various ranks within a sequence. The attention mechanism must jointly use different representation subspaces of queries, keys, and values; the latter are transformed with independently learned linear projections. In the end, the results of the attention grouping are concatenated and transformed with another learned linear forecast to produce the final result, where each of the outputs of the attention clustering is a head, resulting in the design known as multi-headed attention [42]. The model used in this article is shown in the Fig. 6. Equation 4 that describes it is represented taking into account that ‘Q’ is the vector that represents the current token and is used to calculate the following tokens, ‘K’ is the key, and ‘v’ is the value of the vector that contains relevant information. ‘dk’ is an attention normalization constant.

4 $$\begin{aligned} \text {Attn}(\textbf{Q}, \textbf{K}, \textbf{V}) = \text {softmax}\left( \frac{\textbf{Q}\textbf{K}^T}{\sqrt{d_k}}\right) \textbf{V} \end{aligned}$$

### Metrics

Tabares-Soto Et al. explain the relevance of metrics in the evaluation of a model, highlighting the distinction between false positives (FP), false negatives (FN), true positives (TP), and true negatives (TN) [43–45]. The most important metrics are the following:

#### Accuracy

Accuracy is the fraction ranging from 0 to 1, representing the correct prediction percentage. To achieve this metric, the total correct predictions are divided by the total predictions made [44–48] (see Eq. 5).

5 $$\begin{aligned} Accuracy=\frac{TP+TN}{TP+TN+FP+FN} \end{aligned}$$

#### Precision

This metric aims to identify the correct proportion of positive cases, including both false positives and true positives. It is calculated by dividing the number of true positives by the sum of true positives and false positives [44, 45, 48, 49] (see Eq. 6).

6 $$\begin{aligned} Precision=\frac{TP}{TP+FP} \end{aligned}$$

#### Recall

Also known as sensitivity, it shows the ability of the classifier to display correct predictions [44, 45, 48] (see Eq. 7).

7 $$\begin{aligned} Recall=\frac{TP}{TP+FN} \end{aligned}$$

#### F1

F1 is a metric used to assess the model’s ability to accurately identify positive and negative cases. This metric is sensitive to imbalance. It is calculated as the harmonic mean between precision and recall [44, 45, 48, 50] (see Eq. 8).

8 $$\begin{aligned} F1=2x\frac{{Precision\,x\,Recall}}{{Precision + Recall}} \end{aligned}$$

#### Support

This metric indicates the number of data in each test class.

#### Confusion matrix

The confusion matrix is the combination of the actual and predicted classes. The rows represent the envisioned classes, and the columns represent the real class [44, 48].

#### Cross-validation (CV)

Cross-validation is used to evaluate the performance of a model. It divides it into several subsets known as “folds” (k) of similar sizes, generating a process of interactions in the model in which data are obtained at the end of which an average is obtained [44, 48]. In this case, we used “ten folds”. Equation 9 represents it:

9 $$\begin{aligned} \text {Cross-Validation} = \frac{1}{k} \sum \limits _{i=1}^{k} \text {Performance}_i \end{aligned}$$

### Model configuration

#### Convolutional neural network-fully connected (CNNs-Fully)

This is the combination of feature extraction Fig. 3 with a densely connected neural network Fig. 4. The input tensor of the convolutional neural network has a shape of (None, 178, 1), and the input tensor to the Fully connected is (None, 2, 512). This changes when we apply PCA, as the input tensor for the convolutional network becomes (None, 40, 1), and the input to the Fully connected is (None, 1, 512).

#### Convolutional neural network-caps_net (CNNs-capsule-net)

Here we can see the main feature extraction base Fig. 3 connected to the modified capsule for signal reading Fig. 5. The input tensor of the convolutional neural network has a shape of (None, 178, 1), and the input tensor to the Capsule-Net is (None, 2, 512). This changes when we apply PCA, as the input tensor for the convolutional network becomes (None, 40, 1), and the input to the Capsule-Net is (None, 1, 512).

#### Convolutional neural network-transformer encoder (CNNs-Tf)

Characteristic extraction base Fig. 3 followed by the transformer encoder attention model Fig. 6. The input tensor of the convolutional neural network has a shape of (None, 178, 1), and the input tensor to the transformer encoder is (None, 2, 512). This changes when we apply PCA, as the input tensor for the convolutional network becomes (None, 40, 1), and the input to the transformer encoder is (None, 1, 512).

#### Convolutional neural network-transformer encoder-fully connected (CNNs-Tf-fully)

Characteristic extraction base Fig. 3 followed by the transformer care model Fig. 6 and the densely connected network model Fig. 4. The input tensor for the convolutional neural network has a shape of (None, 178, 1), the input tensor for the transformer is (None, 2, 512), and the input tensor for the Fully Connected is (None, 2, 512). This changes when applying PCA, as the input tensor for the convolutional network becomes (None, 40, 1), the input to the Transformer Encoder is (None, 1, 512), and for the Fully Connected, it is (None, 1, 512).

#### Convolutional neural network-transformer encoder and capsule-net (CNNs-Tf-capsule-net)

Mainly the feature extraction layer Fig. 3, followed by the model attention transformer encoder Fig. 6, and finally, the modified capsule Fig. 5. The input tensor for the convolutional neural network has a shape of (None, 178, 1), the input tensor for the transformer is (None, 2, 512), and the input tensor for the Capsule-Net is (None, 2, 512). This changes when applying PCA, as the input tensor for the convolutional network becomes (None, 40, 1), the input to the Transformer Encoder is (None, 1, 512), and for the Capsule-Net, it is (None, 1, 512).

### Hardware and resources

The experiments used Google Colab, where specific computations were performed on the NVIDIA GP100GL [T4 PCIe 15GB] platform, equipped with 250W power, CUDA Version 10.1, and 12 GB of RAM.

## Results

### Iteration hyperparameters of the transformer model

To achieve the best possible results, the hyperparameters of the transformer encoder, such as the attention heads and the layers, were iterated to find the most optimal ones for each variation of the models. In Table 4, we can see the results of each interaction with their respective results, where the best model was the encoder transformer model without any aggregate with 16 attention heads. This was possible because our database does not have a significant computational cost.

**Table 4:** Interaction results on the hyperparameters of the encoder transformer model with its combinations

Algorithms	Numheads	Accuracy [%]	Layers	Accuracy [%]
**CNN+Tf+Capsule-Net**	1	85.26	1	86.74
	2	84.43	2	84.91
	4	84.78	4	86.26
	8	86.91	8	85.70
	16	85.74	16	85.04
**CNN+Tf**	1	86.30	1	86.00
	2	87.22	2	82.30
	4	84.96	4	87.30
	8	87.13	8	86.35
	16	87.57	16	85.39
**CNN+Tf+Fully**	1	86.30	1	87.09
	2	86.57	2	85.43
	4	85.61	4	86.22
	8	85.57	8	84.48
	16	86.74	16	86.22

### Binary classification

In the case of binary classification, whether the patient had epilepsy or not, we have the Table 5, which shows the data results without applying a balancing model. In Table 6, we have the results of the balanced data using SMOTE, and Table 7 have the result of the balanced data using ADASYN. These three tables are divided where the first column is the used model, then we have its accuracy, then its cross-validation, and finally, we have the model’s sensitivity for each class.

With the unbalanced data, we have very high results in most cases. However, it is essential to highlight that the worst model was machine learning, specifically the SGD, with an accuracy of 0.83 and equal cross-validation. However, the model correctly classifies patients with epilepsy; however, in the other classes, it presents a failure. And as a classification model with higher accuracy, we have the transformer encoder model with an accuracy of 0.9974, a cross-validation of 0.998, and an accuracy in both classes of 0.998. It is also important to highlight that the best machine learning model was SVM with an accuracy of 0.9830 and a precision, although close, higher in the case of the data set of patients with the pathology.

**Table 5:** Results of all binary classification models with their respective cross-validation and their accuracy of the unbalanced database

			Epileptic	Without Epileptic
	Accuracy [%]	Cross Validation [%]	Precision [%]	Precision [%]
**ETC**	97.87	97.80 ± 0.5	96.60	98.20
**RFC**	98.13	98.00 ± 1.0	96.30	98.60
**GB**	97.43	97.40 ± 2.0	97.70	97.40
**DTC**	94.09	94.00 ± 1.0	85.70	95.60
**MLP**	98.13	98.00 ± 0.5	96.80	98.10
**KNN**	94.78	95.00 ± 0.4	99.10	94.00
**SGD**	83.87	83.90 ± 1.0	95.70	83.80
**SVM**	98.30	98.00 ± 0.3	96.70	98.70
**CNN+Fully**	99.61	99.00 ± 1.0	99.80	99.90
**CNN+Capsule-Net**	99.35	99.30 ± 1.0	98.10	99.70
**CNN+TF**	**99.83**	99.80 ± 0.3	99.80	99.80
**CNN+Tf+FULLY**	99.74	99.80 ± 1.0	99.60	99.80
**CNN+TF+Capsule-Net**	99.65	99.70 ± 1.0	99.60	99.70

On the other hand, in the balanced database, as in the unbalanced one, the worst model is the machine learning SDG; however, in this case, we have an accuracy of 0.669 and a cross-validation of 0.54, showing that the model is not efficient for this classification problem. The best model was the Capsule-Net model, with an accuracy of 0.992. The best machine learning model was KNN, with a remarkable cross-validation of 1.

**Table 6:** Results of all binary classification models with their respective cross-validation and their accuracy of the balanced database using SMOTE

			Epileptic	Without Epileptic
	Accuracy [%]	Cross Validation [%]	Precision [%]	Precision [%]
**ETC**	98.42	98.40 ± 0.3	97.70	99.10
**RFC**	97.74	98.00 ± 1.0	96.40	99.10
**GB**	98.18	98.20 ± 1.0	97.50	98.80
**DTC**	93.51	93.00 ± 0.5	93.50	93.90
**MLP**	98.59	99.00 ± 0.1	98.00	99.60
**KNN**	99.59	100 ± 0.1	99.60	99.60
**SGD**	66.90	66.50 ± 1.0	93.60	60.50
**SVM**	98.32	98.50 ± 0.3	97.60	99.00
**CNN+Fully**	99.78	99.80 ± 0.1	99.80	99.80
**CNN+Capsule-Net**	**99.92**	99.90 ± 0.4	100	99.80
**CNN+TF**	99.76	99.80 ± 1.0	99.50	100
**CNN+Tf+FULLY**	99.57	99.40 ± 1.0	100	99.20
**CNN+TF+Capsule-Net**	99.89	99.90 ± 0.4	99.80	100

**Table 7:** Results of all binary classification models with their respective cross-validation and their accuracy of the balanced database using ADASYN

			Epileptic	Without Epileptic
	Accuracy [%]	Cross Validation [%]	Precision [%]	Precision [%]
**ETC**	97.87	98.50 ± 1.0	98.90	96.80
**RFC**	97.04	96.00 ± 2.0	99.00	95.20
**GB**	98.18	98.20 ± 1.0	97.50	98.80
**DTC**	89.96	91.00 ± 3.0	89.50	90.40
**MLP**	98.60	98.70 ± 0.1	99.60	97.60
**KNN**	99.81	1.000 ± 1.5	99.60	99.60
**SGD**	61.44	62.50 ± 1.0	58.00	71.90
**SVM**	98.63	98.80 ± 0.5	99.70	97.60
**CNN+Fully**	99.40	99.50 ± 1	99.50	99.30
**CNN+Capsule-Net**	98.22	98.50 ± 0.4	98.40	98.10
**CNN+TF**	94.42	92.80 ± 2.0	90.70	99.00
**CNN+Tf+FULLY**	97.83	96.50 ± 1.0	99.9	97.90
**CNN+TF+Capsule-Net**	98.91	98.20 ± 1.0	99.10	98.70

### Multiclase

Table 8 shows the results obtained from the models evaluated in the five classes of the database. All the models were evaluated in 4 different ways: the first is “Any”, which means the database without any process before entering the network; the second, “Scaling”, refers to the use of Standard Scaler; the third is with the use of PCA and finally the combination of standard scaler and PCA. We can see the hyperparameters used and its result.

**Table 8:** Comparative table of the different models used for comparison. Each of these models was evaluated in 5 classes: Any, scaling, PCA, and scaling + PCA, the hyperparameters used, and, finally, their accuracy is mentioned

Algorithms	Conditions on the dataset	Tuning Hyperparameters	Cross Validation [%]	Accuracy [%]
**DTC**	Any	criterion = gini, min_samples_leaf =2, min_samples_split = 10	48.00 ± 2.0	48.74
	Scaling		48.00 ± 2.0	49.43
	PCA		55.00 ± 2.0	53.61
	Scaling + PCA		54.00 ± 1.0	54.91
**MLP**	Any	activation = relu, hidden_layer_sizes = (100,50), learning_rate = constant, solver = Adam	30.00 ± 1.0	52.65
	Scaling		69.00 ± 1.0	72.39
	PCA		37.00 ± 1.0	59.09
	Scaling + PCA		69.00 ± 1.0	71.22
**KNN**	Any	algorithm = auto, leaf_size = 1, n_neighbors= 1, p = 2, weights = ‘uniform’	56.00 ± 1.0	54.26
	Scaling		56.00 ± 1.0	54.30
	PCA		57.00 ± 1.0	57.65
	Scaling + PCA		57.00 ± 1.0	57.61
**ETC**	Any	n_estimators = 300, random_state = 20, weights = ‘uniform’	72.00 ± 1.0	73.48
	Scaling		72.00± 1.0	73.48
	PCA		75.00 ± 1.0	75.87
	Scaling + PCA		76.00 ± 1.0	76.39
**SVM**	Any	C = 10, gama = ‘scale’, kernel = ‘rbf’	20.00 ± 1.0	19.96
	Scaling		64.00 ± 1.0	64.30
	PCA		19.00 ± 1.0	20.70
	Scaling + PCA		70.00 ± 1.0	71.09
**RFC**	Any	max_depth = None, min_samples_split = 2, n_estimators = 500, random_state = 40	73.00 ± 1.0	73.26
	Scaling		73.00 ± 1.0	73.22
	PCA		74.00 ± 1.0	73.48
	Scaling + PCA		74.00 ± 2.0	73.39
**GB**	Any	learning_rate = 0.1, max_depth = 7, n_estimators = 200, random_state = 10	69.00 ± 1.0	69.22
	Scaling		0.69 ± 1.0	69.26
	PCA		72.00 ± 1.0	72.61
	Scaling + PCA		71.00 ± 1.0	71.74
**CNN+Fully**	Any	optimizer=Adam(lr=0.001), epochs=500, batch_size=128	79.00 ± 2.0	83.83
	Scaling		85.00 ± 1.0	85.04
	PCA		55.00 ± 4.0	60.52
	Scaling + PCA		72.00 ± 3.0	72.04
**CNN+Capsule-Net**	Any	num_caps = 16, optimizer=Adam(lr=0.001), epochs=500, batch_size=128	79.00± 2.0	73.30
	Scaling		86.00 ± 1.0	**87.13**
	PCA		56.00 ± 3.0	56.65
	Scaling + PCA		72.00 ± 3.0	74.30
**CNN+Tf**	Any	NUM_HEADS = 16, NUM_LAYERS= 2, epochs=500, batch_size=128	79.00 ± 2.0	74.48
	Scaling		86.00 ± 2.0	**88.34**
	PCA		48.00 ± 1.0	49.35
	Scaling + PCA		72.00 ± 2.0	67.39
**CNN+TF+Fully**	Any	NUM_HEADS = 16, NUM_LAYERS= 1, epochs=500, batch_size=128	75.00 ± 1.0	76.74
	Scaling		85.00 ± 1.0	85.91
	PCA		57.00 ± 2.0	56.22
	Scaling + PCA		75.00 ± 1.0	75.00
**CNN+Tf+Capsule-Net**	Any	NUM_HEADS = 8, NUM_LAYERS = 1,num_caps = 16, epochs=500, batch_size=128	79.00 ± 2.0	80.83
	Scaling		86.00 ± 1.0	85.09
	PCA		53.00 ± 2.0	57.78
	Scaling + PCA		71.00 ± 2.0	72.35

**Fig. 7:**
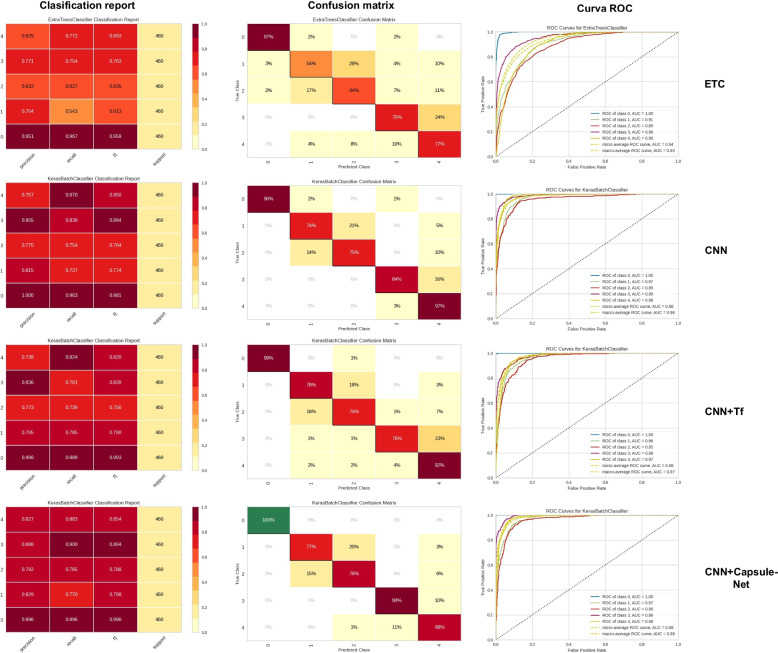
Comparative table of the metrics of the best model for multi-class classification of machine learning, the CNN, the tf, and the caps-net. In this one, we can see the first column, the classification report, the second, the confusion matrix, and the third, the ROC curves

On the other hand, Fig. 7 we have the comparison between the models where the best machine learning model, the extra tree classifier, presents the lowest result; however, its accuracy in the class of patients with an epileptic seizure stands out, but the best model is the Capsule-Net, as in the binary model, where we have the highest accuracy in all classes and a more leveled confusion matrix.

### Time of compilation

**Fig. 8:**
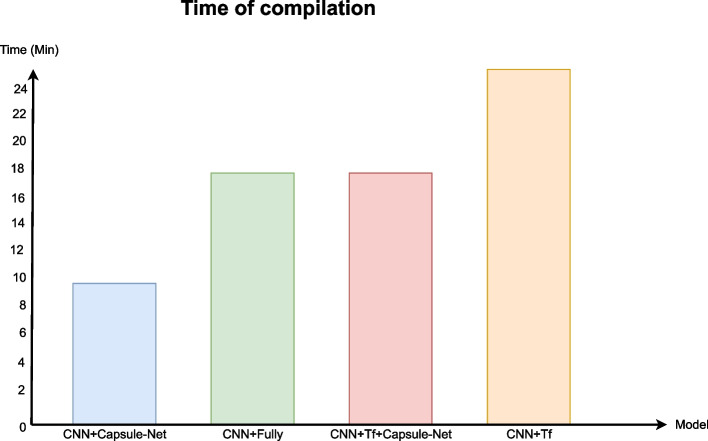
Bar chart of the compilation times in minutes of the four best models

As the results of the best models are so close, it is essential to analyze other variables, such as compilation time and computational resource expenditure. Figure 8 shows a bar chart comparing the compilation times of the best models, where it is evident that the model that takes less time to compile for 500 epochs is the Capsule-Net model, the fully connected and Transformer Encoder+Capsule-Net models have the same time and the most delayed is the transformer encoder model. The compilation time is calculated during training by multiplying the duration of each epoch by the total number of epochs and finally dividing by 60 to convert from seconds to minutes.

### Comparison with the state of the art

The results are compared with the state-of-the-art using the same database and division. This can be seen in Table 9.

**Table 9:** Comparison of the accuracy in relation to the state of the art

Algorithms	Authors	Multiclass accuracy	Binary accuracy	Time of compilation [Min]	Parameters [millions]
1D-CNN-LSTM	Gaowei Xu et al. [12].	82.00	99.39	266	2.23
**CNN+Capsule-Net**	**Proposed model**	**87.00**	**99.92**	**8**	5.04
**CNN+Tf**	**Proposed model**	**88.00**	**99.76**	25	22.82

### Gradient-weighted class activation mapping (GradCam)

**Fig. 9:**
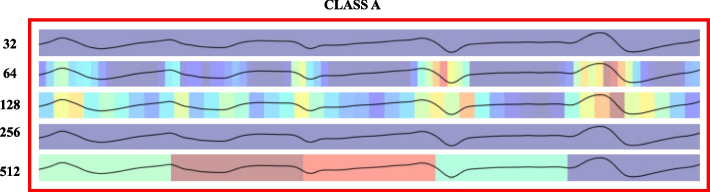
GradCam of the EEG from class (a), i.e., patients with an epileptic seizure, in each convolutional layer 32, 64, 128, 256, 512 that can be seen on the y-axis

**Fig. 10:**
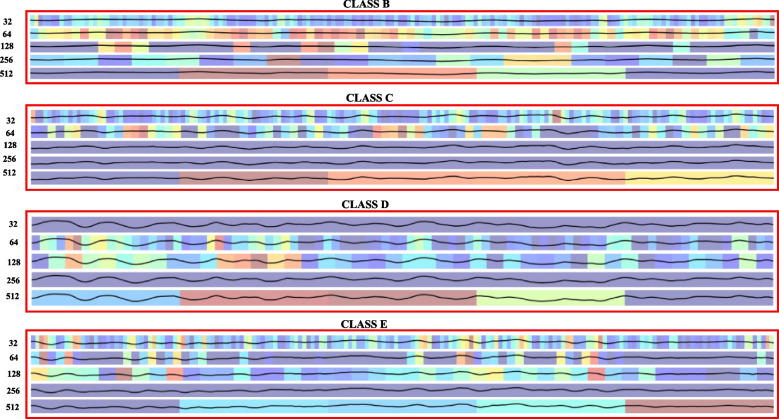
GradCam of the EEG from classes (b), (c), (d), (e), i.e., patients without an epileptic seizure, in each of the convolutional layers 32, 64, 128, 256, 512 that can be seen on the y-axis

GradCam, this method interprets convolutional neural network models by visually presenting the input regions the model deems most crucial for making predictions. It relies on calculating the gradient of the predicted class score concerning the feature maps of the final convolutional layer. These maps are then globally averaged to derive weights multiplied by their respective inputs, resulting in a map highlighting the importance of the input variable [51]. The Grad-CAM for this issue can be observed in Fig. 9, which pertains to patients with epileptic seizures, and Fig. 10 illustrates the remaining patients.

### Feature importance

**Fig. 11:**
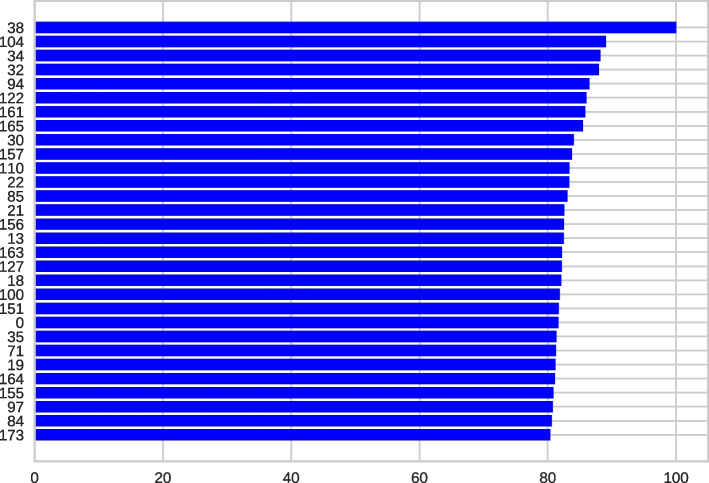
The 30 most crucial points in the EEG, representing the key features for the ETC (Event-Triggered Control)

Understanding the significance of features is a fundamental technique in interpreting ML models. It enhances our comprehension of the model’s functioning and assists in recognizing biases and crucial features. This approach is essential as artificial intelligence models have grown progressively complex and challenging to interpret, mainly owing to scientific advancements [52] (see Fig. 11).

## Discussion

Epilepsy is a severe disease that, due to lack of knowledge, has been cataloged as a taboo and considered less important than it is, worsening the patient’s quality of life and even causing death. However, an accurate and quick treatment can help the patient have a relatively everyday life, so it is essential to use new technologies to achieve a more efficient process.

It is common to use an electroencephalogram for diagnosing pathology since its cost can be meager compared to other medical imaging methods. This opens the possibility of using artificial intelligence methods with electroencephalogram databases. However, there are very few public databases, so it is necessary to explore all options. In this case, different ways of processing the database were used to find the best result by evaluating them in other traditional and state-of-the-art models.

For traditional machine learning models, their efficiency is directly related to the tuning of their hyperparameters. Here is where the pipeline and grid search algorithms that we observe in Table 2 are essential to efficiently search for the best combination for the evaluation of each of the database partitions observed in Figs. 1 and 2.

As shown in Fig. 1, the model of two classes, i.e., of patients with epilepsy against the rest of the categories, is unbalanced. To correct this problem, we used synthetic data using algorithms such as SMOTE and ADASYN in the tuning of hyperparameters in the ML models we can see in Table 3 that the hyperparameters for each of the types of data balancing do not have a natural significant variation, so it can be decided to use one or the other.

Directly in the state-of-the-art algorithms, we can observe in the “Models” section and in the “Model configuration” section, it is evident that they all have feature extraction using CNN as their origin. Experiments obtained the best layers and activation methods. In the case of convolutional layers, we can observe in Fig. 3 the most efficient activation method for this database is selu. Modifying the capsule initially designed for reading images generates an essential contribution to the state of the art. It is necessary to emphasize that according to the author of the original capsule, the max pool can generate problems and worsen the accuracy. In the case of these signals, we have an improvement when using the global max pooling, possibly because we are working with signals and not with images.

Continuing with state-of-the-art, as briefly mentioned in the “Introduction” section, the combination of models has been the latest trend for classification problems; however, the state-of-the-art does not report combinations associated with this pathology, nor to this specific database, experimentation with the variety of models to use the best features of each one is an important contribution presented in this paper.

Now focusing directly on the results obtained in this article, Table 4 shows the interaction of the hyperparameters of the encoder transformer model with its different variations. It is essential to highlight that by using flat data, the expenditure in computational resources is not so high, which makes it possible to increase the number of heads of attention and experimentation without having problems due to the lack of powerful graphics cards.

In binary classification based on whether the patient has epilepsy or not, we can observe that, although state-of-the-art models show higher efficiency, there is no significant difference among all models based on their standard deviation or cross validation of unbalanced data, as seen in Table 5. This difference is much smaller when the data is balanced, as shown in Tables 6 and 7. However, the metrics improve when we have balanced classes. This indicates that synthesized data effectively increases the system’s signal recognition capacity. Among the balancing methods, SMOTE and ADASYN, SMOTE proves to be more efficient and achieves better metrics, achieving an accuracy of 99.92% in the capsule net model and 99.59% in the KNN model. However, since they are synthetic data, validating again with data from another database is necessary.

Entering directly into the five database classes, we can observe in Fig. 2. As mentioned at the beginning of the discussion, database processing plays a fundamental role in finding the best model. As seen in Table 8, processing the database is necessary since the unprocessed database yields meager results, ranging from 20% to 69% in ML models and from 76% to 88% in DL models. Analyzing the variability of PCA components does not show an improvement in the results obtained. This may be because each point in the EEG contributes information and variability to the model. When analyzing the results in ML models using PCA, we obtain results ranging from 19% to 75%. Despite SVM presenting the worst results, applying PCA in DL models improves the performance of ML models and demonstrates superior metrics.

Now, standard scaling is the one that best fits the majority of the database, where we see that both ML and DL models show a significant improvement in their metrics, except for some cases like ETC, where the best option is to apply PCA and standard scaling. However, machine learning models could be more efficient for classifying the five classes in this problem, with the best results observed in decision tree models like ETC with 73.48% accuracy. Nevertheless, they need to catch up compared to DL models such as CNN+TF, which shows 88.34% accuracy, or the CNN+Capsule-Net model, which shows 87.13% accuracy, with lower standard deviation and compilation time. In this case, individual models perform better than combining them, as in the case of CNN+TF+Capsule-Net, which proves to be inferior to the personal evaluation of each one, achieving only 85.09% accuracy.

In Fig. 7, where we can compare the best ML and state-of-the-art models, we can see that the state-of-the-art models are much more efficient. However, a constant is demonstrated in all models, and EEGs related to brain tumors, classes b and c, have a problem with classification.

In addition to the above, we can use GradCam from the convolutional layers for better interpretability, as shown in Figs. 9 and 10. These visually depict the waveform and its behavior in the final or convolutional layers. Additionally, we can observe the feature extraction graph in Fig. 11, where graphically, it is evident that most of the features or points in the EEG are crucial for classification. This may be a reason why PCA does not yield good results.

Finally, looking for the best model, the difference between the two best models, CNNs+Capsule-Net and CNNs+Transformer Encoder, is only one percentage point. Still, the Capsule-Net has a lower standard deviation, achieving a more stable result. Besides that, Fig. 8 shows that the compilation time and, consequently the computational resource expenditure when using CNNs+Capsule-Net is three times less than when using the CNNs+Transformer Encoder model concluding that the best classification model is the proposed CNNs+Capsule-Net model modified for signals achieving an accuracy of 87.30% and a standard deviation of ± 1%. However, the state-of-the-art is surpassed with both models, Furthermore, the compilation time is significantly shorter, even with more parameters. This is due to the efficiency of the models and their parallel processing, unlike LSTMs that operate sequentially as shown in Table 9.

The creation of diagnostic tools with the help of artificial intelligence is an innovative field in medical technology. Tools such as classification models applied to medical services can make treatments more assertive and faster, as doctors would have an additional tool to confirm or reject a diagnosis. This can reduce time-consuming and costly processes in developing countries, impacting the directly affected users. Artificial intelligence and diagnostic tools have the potential to save lives.

## Conclusion

The use of artificial intelligence models for diagnosing pathologies has experienced significant growth in the last decade, making it essential to find the model that best suits each studied disease. In the case of epilepsy using electroencephalographic signals, the difference between models is minimal, so it is crucial to analyze other aspects, such as computational resource expenditure and compilation time. Regarding machine learning models, compilation times are minimal due to their high optimization. However, the results are much lower when classifying multiple types of electroencephalograms, possibly due to the reduced data. The metrics are significantly lower. The ML models, such as ETC or RFC, generally show the best results for this issue. Specifically, concerning state-of-the-art models, it can be concluded from this analysis that the best model for classifying electroencephalograms is the CNNs+Capsule-Net model. It achieves accuracy only one percentage point below the transformers model but with a lower standard deviation and, most importantly, half the compilation time.

### Suggestions for future research

For future work, evaluating the proposed models with new data and different signal acquisition methods is advisable to verify their suitability for deployment in a hospital setting. This is why a repository with the codes is included so that experiments can be replicated and eventually improved.

## Data Availability

The datasets and code used during the current study are available at: https://github.com/BioAITeam/A-Comparative-Study-of-CNN-Capsule-Net-CNN-Transformer-Encoder-and-traditional-Machine-Learning-Al.

## Abbreviations

EEG: Electroencephalogram
CNNs: Convolutional neural networks
Capsule-Net: Capsule neural networks
ML: Machine learning
ViT: Vision transformer
LSTM: Long short-term memory network
DL: Deep learning
ETC: Extra Trees Classifier
RFC: Random Forest Classifier
SVM: Super Vector Machine
GB: Gradient Boosting
DTC: Decision Tree Classifier
KNN: Kneighboors Classifier
SGD: Stochastic Gradient Descent
Fully: Fully Conected
Tf: Transformer Encoder
